# Who, When, and How: Watchful Waiting in the ERSPC Rotterdam

**DOI:** 10.1016/j.euros.2026.03.013

**Published:** 2026-04-02

**Authors:** Jeroen J. Lodder, Esmée F.H. Mulder, Sebastiaan Remmers, Roderick C.N. van den Bergh, Chris H. Bangma, Monique J. Roobol, ERSPC Rotterdam Study Group, W.J. Kirkels, J.B.W. Rietbergen, I.W. van der Cruijsen, R. Raaijmakers, S.H. de Vries, S. Roemeling, C. Gosselaar, T. Wolters, R.C.N. van den Bergh, P.J. van Leeuwen, M. Bul, X. Zhu, L.P. Bokhorst, A.R. Alberts, F-J. Drost, J.F.M. Verbeek, D.F. Osses, H.B. Luiting, R. Hogenhout, R.C.A. Leenen, J.J. Lodder, M.J. van Harten, S.F. Westerhout, F. Denijs, E.F.H. Mulder, L.D.F. Venderbos, K. Beyer, H.A. van Vugt, G. Yurdakul, A. Boeken-Kruger, C. Wijburg, M. Forouzanfor, M. de Boer, R. Postma, A.N. Vis, Th van der Kwast, R. Hoedemaeker, A. van Leenders, R. van Schaik, P.J. van der Maas, H.J. De Koning, E.A. Heijnsdijk, S. Otto, G. Draisma, P. Beemsterboer, I. Korfage, R. Boer, M. Wildhagen, D. Nieboer, W. Merkelbach, W. Hoekstra, J. Blom, T. Lock, A. Noordzij, RAM Damhuis, A. Reedijk, R. Kranse, D.W. Roobol, W. Roobol, E. van den Berg, G-J. de Zwart, C.G.A.M. Franken-Raab, M. van Slooten-Midderig, A. Smit, V. van der Drift, E. de Bilde, M. den Rooijen, L. Mani, M. Visser-van Dongen, H. Versteeg-Leenheer, B. Zoutendijk, H. van Meurs, A.E. de Bruijn

**Affiliations:** Department of Urology, Erasmus MC Cancer Institute, Erasmus University Medical Center, Rotterdam, The Netherlands

**Keywords:** *A*ndrogen deprivation therapy, Prostate cancer, Risk stratification, Watchful waiting

## Abstract

**Background and objective:**

Long-term data identifying which patients managed with watchful waiting (WW) ultimately require androgen deprivation therapy (ADT) are limited. Using 18-yr follow-up data from the Rotterdam section of the European Randomized Study for Prostate Cancer, we examined WW practices and identified prognostic factors for ADT initiation.

**Methods:**

Men whose initial management strategy was WW were included and stratified by European Association of Urology risk group. WW practices were evaluated by the frequency of follow-up visits, prostate-specific antigen (PSA) testing, and imaging. Prognostic factors for ADT initiation were identified using a Fine-Gray competing risk model and used to predict 18-yr ADT-free survival for three hypothetical patients representing the risk groups.

**Key findings and limitations:**

We included 537 men with a median follow-up of 12.9 yr (interquartile range: 7.5–16.7). At 18 yr, cumulative incidence of ADT initiation was 6.6% (95% confidence interval [CI]: 0.02–13), 33% (95% CI 26–39), and 55% (95% CI 47–64) in men at low, intermediate, and high risk, respectively. Most patients received regular follow-up every 6 mo, regardless of risk group, whereas imaging was uncommon except in men at higher risk. PSA, grade group, and clinical T-stage were statistically significant predictors of ADT initiation. Predicted 18-yr ADT-free survival ranged from 90% in men at low risk to 35% in men at high risk. Limitations include the retrospective design, use of ADT initiation as an end point without standardized triggers, and a historical cohort, possibly affecting internal validity and generalizability.

**Conclusions and clinical implications:**

Follow-up for men managed with WW could be individualized based on patient and tumor characteristics to reduce unnecessary visits while preserving timely detection of progression.

## Take Home Message

Patients with intermediate- and high-risk prostate cancer managed with watchful waiting are more likely to require androgen deprivation therapy, and therefore, benefit more from monitoring. A risk-adapted follow-up strategy can safely reduce visits for men at low risk while ensuring timely intervention for those at higher risk.

ADVANCING PRACTICE **What does this study add?**

This study assessed how men with prostate cancer managed by watchful waiting are monitored in clinical practice and which factors predict the need for androgen deprivation therapy. These findings may support clinicians in tailoring follow-up intensity based on patient- and tumor-specific characteristics, thereby reducing unnecessary follow-up visits.

**Clinical Relevance**

In this analysis, based on the Rotterdam section of ERSPC, the long term outcome after 18 years of follow-up in patients undergoing watchful waiting is reported. The need of ADT in these patients is heavily driven by upfront EAU risk group status, which might lead to risk adapted management strategies in accordance to upfront clinical characteristics. Associate Editor: Jochen Walz.

**Patient Summary**

We studied how men with prostate cancer who were managed by watchful waiting were monitored and which men later required hormone therapy. Most men had regular checkups, but only those with higher-risk disease often received additional tests or treatment. Our findings support a more personalized approach to follow-up—less frequent for men at low risk and more frequent for men at higher risk—to reduce unnecessary visits while ensuring timely care.

## 1 Introduction

Watchful waiting (WW) is a conservative management strategy for men with nonmetastatic, asymptomatic prostate cancer (PCa) who are considered unsuitable for or unlikely to benefit from curative treatment, typically because of a combination of advanced age, limited life expectancy, and/or low risk of clinically significant tumor progression [1]. Unlike active surveillance (AS), which involves close monitoring of patients eligible for curative treatment, WW traditionally involves less strict follow-up and defers intervention until symptoms develop, at which point palliative treatment, most often androgen deprivation therapy (ADT), can be initiated [2].

Historically, WW entailed no routine monitoring, and ADT was initiated only in response to symptomatic local or metastatic progression [2], with symptoms ranging from lower urinary tract obstruction to bone pain. Such symptoms could arise unexpectedly and lead to acute complications, such as urinary retention, pathological fractures, or spinal cord compression [3]. Concerns about unpredictable disease trajectories, together with evidence suggesting a survival benefit from earlier ADT initiation in selected patients at higher risk [4], often lead to some degree of scheduled follow-up in many men managed with WW in clinical practice. However, the evidence supporting early ADT is limited and context-dependent. In the Scandinavian Prostate Cancer Group (SCPG)-6 trial, comparing ADT to placebo in 1218 hormone naive, nonmetastatic patients with PCa, an overall survival (OS) benefit was seen only in high-risk, fast-progressing patients [4]. Similarly, a randomized controlled trial of 234 patients with node-positive PCa (pN1–3) found no OS benefit when comparing immediate versus delayed hormonal treatment [5]. Importantly, ADT has well-documented adverse effects, including reduced quality of life [6] and increased cardiovascular and metabolic risk [7], emphasizing the need for careful timing and patient selection.

All major guidelines acknowledge WW as an appropriate management strategy for patients with a life expectancy of <10 yr [1], [8], [9] but provide little guidance on its practical implementation, such as monitoring frequency (if any), use of imaging, or triggers for starting ADT. As a result, clinical practice for men on WW may vary widely [10], [11], [12]. This variation likely reflects the limited evidence on predictors for clinically relevant progression in WW populations. A better assessment of these predictors could support tailored follow-up strategies, helping to avoid both undertreatment (leading to symptomatic progression) and overtreatment (leading to adverse effects).

To address these gaps, we analyzed 18-yr follow-up data after starting WW from the Rotterdam section of the European Randomized Study of Screening for Prostate Cancer (ERSPC). We present WW practices over time, including patterns of PSA monitoring, use of imaging, and ADT-initiation rates, and we identify prognostic factors for ADT initiation.

## 2 Patients and Methods

### 2.1 Data sources and study population

We used data from the Rotterdam section of the ERSPC trial, which randomized men to either a screening or a control arm. Study protocols have been published previously [13], [14], [15]. We included all men (from both arms) who were diagnosed with PCa and whose initial management was recorded as conservative. Because AS was not recognized as a separate treatment strategy when the ERSPC started, disease management was labeled as conservative, meaning either AS or WW, without distinguishing between the two. To enable an evaluation specifically of WW, we retrospectively applied a series of predefined criteria in successive steps to identify the patients managed with the following strategy:

1. First, all men with International Society of Urologic Pathology (ISUP) grade group (GG) ≥2 at diagnosis were assigned to the WW group, as only men with ISUP GG1 were eligible for AS during the ERSPC study period; eligibility for ISUP GG2 for AS was introduced later [16].
2. Among men with ISUP GG1, those who underwent curative treatment or underwent at least one follow-up biopsy during the available follow-up were excluded from the WW group and assigned to the AS group, as these interventions are rarely indicated in WW.
3. Remaining men who did not meet the historical inclusion criteria for AS (ie, PSA <10 ng/ml, clinical T-stage [cT-stage] <T2c, or aged <80 yr at diagnosis) were also assigned to the WW group.
4. Finally, men without regular follow-up visits (defined as PSA measurements and digital rectal exams concordant with the Prostate Research International Active Surveillance protocol ± 1 mo [www.prias-project.org]) were assigned to the WW group. All remaining men were assigned to the AS group.

Follow-up was truncated at 18 yr after diagnosis or by December 31, 2022, whichever came first.

### 2.2 Outcomes

Patients were stratified according to the European Association of Urology (EAU) risk groups for PCa [1]. ADT initiation, other cause mortality (OM), and PCa-specific mortality (PCSM) were calculated for each risk group. ADT initiation was defined as the start of any medical ADT or surgical orchidectomy. The specific clinical indications for initiating ADT (eg, PSA progression, imaging findings, and symptoms) were not available in the dataset. Therefore, ADT initiation was chosen as the most feasible and clinically relevant end point, as it reflects disease progression and is reliably documented in medical records.

WW practices were assessed by analyzing the frequency of follow-up visits, PSA measurements, and imaging (ie, bone scans and computed tomography scans). We calculated the time until the first event and the interval between the first and second events. Patients without any follow-up the first year after diagnosis (with 1 mo margin) were considered not to have received standard follow-up and were excluded from the WW-practices analysis.

Lastly, prognostic factors for ADT initiation were identified, and we used those factors to predict the 18-yr ADT-free survival for three hypothetical patients. These patients were deliberately chosen to resemble the profiles of men in the three EAU risk groups. Comorbidities were registered using self-reported questionnaires.

### 2.3 Statistical analyses

Descriptive statistics were used to report baseline characteristics for each risk group. Cumulative incidences (CIN) were calculated for OM (competing risk: PCSM), PCSM (competing risk: OM), and ADT initiation (competing risk: death before ADT). Patients were first analyzed according to the EAU risk groups, as this classification provides a clinically relevant framework based on PSA, cT-stage, and ISUP GG. However, previous work has shown that additional insights can be gained by considering the role of the individual predictors within these groups [17]. Therefore, we also fitted a Fine-Gray competing risk regression model predicting ADT initiation, using age, PSA, cT-stage (T1, T2, and T3–4), ISUP GG (GG1, GG2, GG3, and GG4–5), and number of comorbidities (0, 1, 2, and ≥3) at diagnosis.

Nonlinearity of continuous variables (age and PSA) was evaluated using restricted cubic splines. Missing data were imputed ten times by multiple imputation using chained equations (MICE), generating ten imputed datasets. For variables with missing values (PSA, cT-stage, and ISUP GG), imputation was performed using the other available variables (age, the remaining tumor characteristics, and number of comorbidities) as predictors. The Fine-Gray model was fitted to each imputed dataset, and estimates were pooled using Rubin’s rule. The pooled model was used to predict ADT-free survival for three hypothetical patients aged 70 yr with one comorbidity, each representing one of the EAU risk groups: a low-risk patient with ISUP GG1, PSA 5 ng/ml, and cT1; an intermediate-risk patient with ISUP GG2, PSA 10 ng/ml, and cT2; and a high-risk patient with ISUP GG4, PSA 15 ng/ml, and cT3.

A sensitivity analysis was performed to assess the influence of PSA doubling time (PSA-DT) within the first year on ADT initiation. PSA values from the first year after diagnosis were used to calculate the PSA-DT, with missing values imputed using MICE for men with only one measurement. Men who died or initiated ADT within the first year were excluded. PSA-DT was calculated as the log of 2 divided by the slope of a linear regression using the log PSA values and time of the measurements for each patient [18]. The index time of this analysis started 1 yr after diagnosis. All analyses were performed in R version 4.3.2 (R Foundation for Statistical Computing, Vienna, Austria).

## 3 Results

**Fig. 1:**
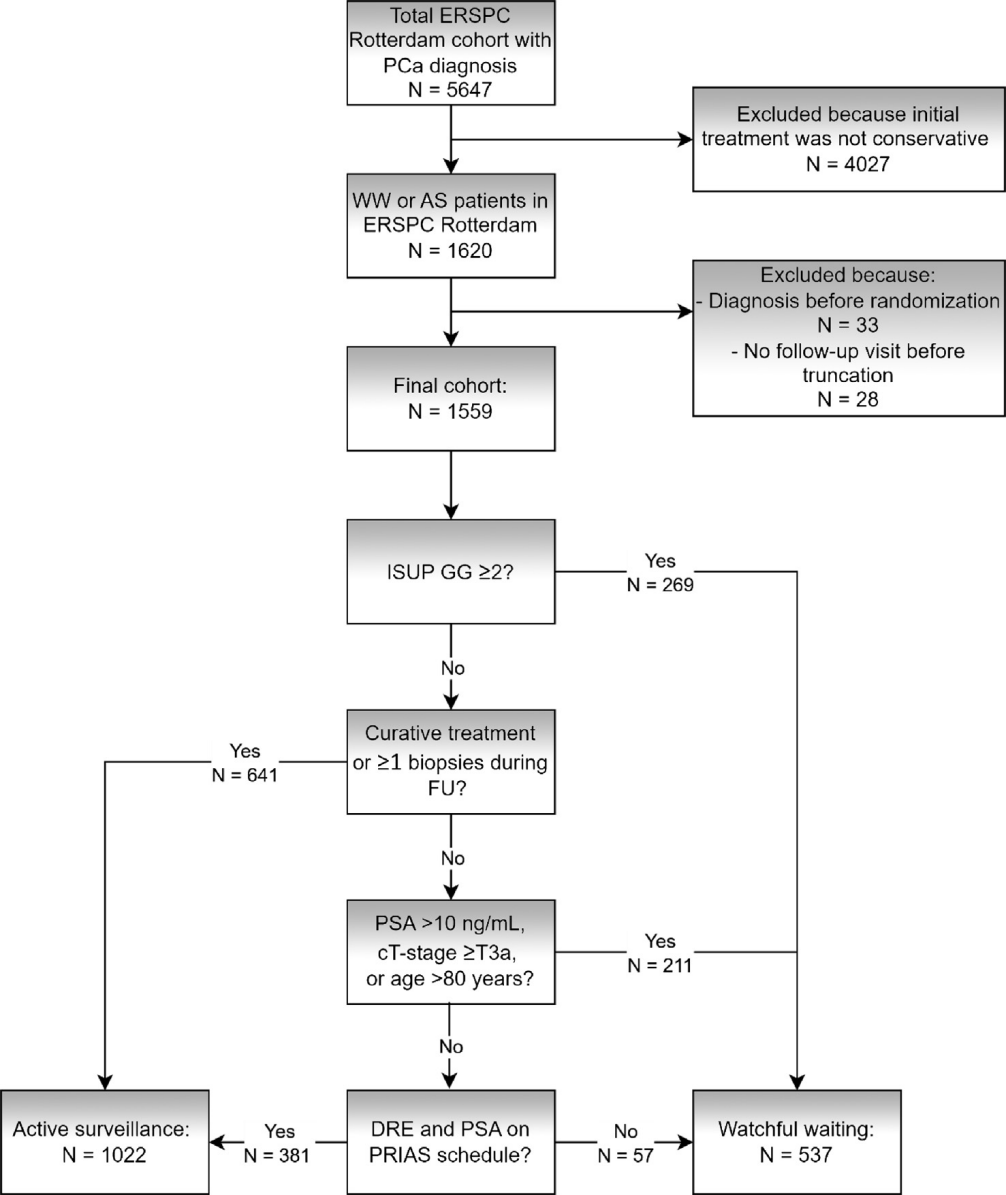
**Patient selection from the ERSPC Rotterdam cohort into either WW or AS.** AS = active surveillance; cT-stage = clinical T-stage; DRE = digital rectal exam; ERSPC = European Randomized Study of Screening for Prostate Cancer; FU = ; ISUP GG = International Society of Urologic Pathology Grade Group; PCa = prostate cancer; PRIAS = Prostate Research International Active Surveillance; PSA = prostate-specific antigen; WW = watchful waiting.

Fig. 1 shows patient selection from the ERSPC Rotterdam cohort into this analysis. After applying the predefined criteria to distinguish between the two conservative management strategies, 1022 patients were assigned to the AS group and 537 to the WW group, which is the main group examined in this analysis. Baseline characteristics of both groups are presented in Supplementary Table 1 and support the applied criteria. Table 1 summarizes patient characteristics of the patients managed with WW, stratified by EAU risk group. Median age at diagnosis was comparable across groups. At 18 yr after diagnosis (median follow-up for men still at risk 12.9 yr, IQR: 7.5–16.7), OM was the highest among men at low risk and lowest in men at high risk. In contrast, PCSM and ADT initiation increased with risk category (Table 1; Supplementary Fig. 1).

**Table 1:** Patient characteristics stratified by EAU risk group

	Low risk (*n* = 76)	Intermediate risk (*n* = 252)	High risk (*n* = 188)
Age (yr)			
Median (IQR)	81 (75–83)	78 (73–81)	80 (76–84)
PSA (ng/ml)			
Median (IQR)	5.0 (2.9–7.1)	11 (5.6–14)	27 (15–47)
Missing	0	5	3
cT-stage, *n* (%)			
T1a	39 (51)	190 (75)	72 (40)
T2	37 (49)	62 (25)	42 (24)
T3	0 (0)	0 (0)	55 (31)
T4	0 (0)	0 (0)	8 (4.5)
Missing, *n*	0	0	10
ISUP GG, *n* (%)			
1	76 (100)	97 (38)	48 (31)
2	0 (0)	119 (47)	34 (22)
3	0 (0)	36 (14)	12 (7.7)
4	0 (0)	0 (0)	36 (23)
5	0 (0)	0 (0)	26 (17)
Missing, *n*	0	0	32
Comorbidities, *n* (%)			
0	26 (34)	88 (35)	55 (29)
1	29 (38)	87 (35)	50 (27)
2	13 (17)	49 (19)	44 (23)
3+	8 (11)	28 (11)	39 (21)
ADT initiation^a^			
CIN (95% CI)	6.6% (0.02–13)	33% (26–39)	55% (47–64)
OM^a^			
CIN (95% CI)	84% (71–97)	71% (63–78)	56% (47–65)
PCSM^a^			
CIN (95% CI)	6% (0–13)	11% (5.9–16)	36% (27–45)

CIN = cumulative incidence; cT-stage = clinical T stage; EAU = European Association of Urology; IQR = interquartile range; PSA = prostate-specific antigen; ISUP GG = International Society of Urological Pathology Grade Group; ADT = androgen deprivation therapy; OM = other cause mortality; PCSM = prostate cancer–specific mortality.

a At 18 yr.

### 3.1 WW practices

In total, 64 patients (12%) received WW without any follow-up measurements registered during the first year after diagnosis (seven low-risk, 29 intermediate-risk, 22 high-risk, six unknown). The remaining men (*n* = 473; 88%) did receive some kind of standard follow-up. Across all groups, median time to first PSA measurement after starting WW was approximately 6 mo, followed by intervals of 6–8 mo. First imaging was performed later—after 23–84 mo—with similar intervals thereafter, and was mainly performed in the intermediate- and high-risk groups (Table 2). The estimated number of follow-up visits needed for one patient to start ADT (number needed to see) was 132, 22, and nine for the low-, intermediate-, and high-risk groups, respectively.

**Table 2:** Timing of follow-up visits and assessments during WW by EAU risk group. Values represent median months (IQR) unless otherwise specified

	**Low risk**	**Intermediate risk**	**High risk**
**A. Time until first follow-up**			
Visit	6.1 (4.4–7.8)	6.5 (4.7–7.8)	6.6 (4.8–7.6)
PSA measurement	6.3 (4.6–8.3)	6.3 (4.2–7.4)	5.8 (4.0–7.7)
Bone scan	36 (29–96)	30 (14–60)	24 (12–42)
CT scan	84 (48–119)	48 (18–88)	23 (12–48)
**B. Time between subsequent assessments, if present**			
Visit	7.1 (6.2–11)	6.7 (6.2–9.0)	6.3 (5.9–7.6)
PSA measurement	8.5 (6.5–12)	7.1 (6.3–11)	6.5 (6.0–8.1)
Bone scan	6.1 (6.1–6.1)	7.8 (6.2–9.7)	7.7 (6.4–8.3)
CT scan	6.0 (6.0–6.0)	6.9 (6.1–10.4)	6.1 (5.2–6.7)
**C. Imaging overview**			
Total bone scans, *n*	5	80	101
Patients with ≥1 bone scan, *n* (%)	2 (2.9)	35 (16)	37 (22)
Positive bone scans, *n* (%)	0 (0)	12 (15)	22 (22)
Total CT scans, *n*	4	62	56
Patients with ≥1 CT scan, *n* (%)	3 (4.3)	24 (11)	24 (14)
Positive CT scans, *n* (%)	1 (25)	10 (16)	19 (34)

CT = computed tomography; IQR = interquartile range; PSA = prostate-specific antigen; WW = watchful waiting; EAU = European Association of Urology.

### 3.2 Prognostic factors for ADT initiation

Nonlinearity did not improve the model’s fit (age: *p* = 0.6; PSA: *p* = 0.2); therefore, it was not included. In the multivariable competing risk regression (Table 3), higher PSA, cT2, and higher ISUP GG were statistically associated with an increased risk of ADT initiation. Conversely, men with ≥2 comorbidities had a statistically significantly lower risk of ADT initiation compared to those without comorbidities. cT3–4 did not reach statistical significance. The sensitivity analysis did not show that PSA-DT was statistically associated with ADT initiation (data not shown).

**Table 3:** Fine-Gray competing risk regression for factors associated with ADT initiation

**Variable at diagnosis**	**sHR (95% CI)**	***p* value**
**Age (from 60 yr)**	0.99 (0.98–1.02)	0.9
**PSA (per doubling)**	1.31 (1.18–1.46)	<0.001
**cT-stage**		
T1	Reference	
T2	1.69 (1.18–2.42)	0.005
T3–T4	1.52 (0.96–2.41)	0.078
**ISUP GG**		
1	Reference	
2	2.84 (1.85–4.35)	<0.001
3	3.79 (2.20–6.53)	<0.001
4–5	5.84 (3.58–9.52)	<0.001
**Comorbidities**		
0	Reference	
1	0.78 (0.53–1.14)	0.2
2	0.60 (0.38–0.95)	0.03
3+	0.58 (0.33–0.99)	0.046

ADT = androgen deprivation therapy; CI = confidence interval; cT-stage = clinical T stage; ISUP GG = International Society of Urological Pathology Grade Group; PSA = prostate-specific antigen; sHR = subdistribution hazard ratio.

### 3.3 Estimated ADT-free survival

**Fig. 2:**
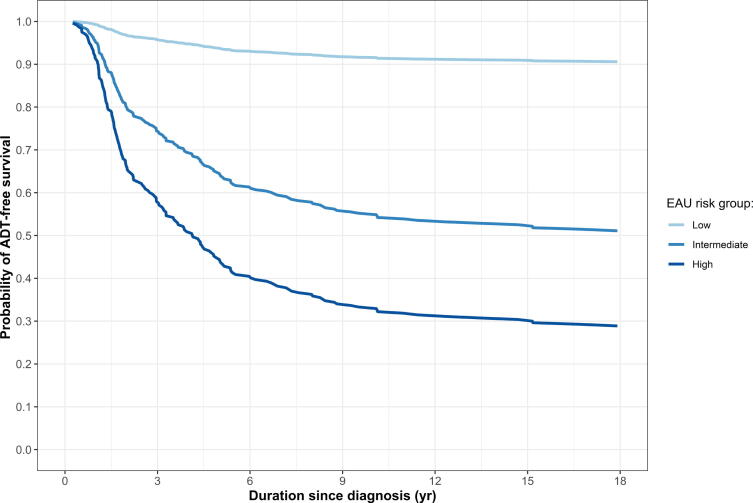
**Probability of ADT-free survival over time for three hypothetical patients with varying characteristics corresponding to the EAU risk groups.** ADT = androgen deprivation therapy; EAU = European Association of Urology.

The model’s predictions for the three hypothetical patients are shown in Fig. 2. Predicted ADT-free survival probabilities at 18 yr were 90% for the patient at low risk (ISUP GG1, PSA 5 ng/ml, and cT1) compared to 52% for the patient at intermediate risk (ISUP GG2, PSA 10 ng/ml, and cT2) and 35% for the patient at high-risk (ISUP GG4, PSA 15 ng/ml, and cT3).

## 4 Discussion

In this study, we analyzed long-term follow-up data from 537 men with nonmetastatic, asymptomatic PCa in the Rotterdam arm of the ERSPC trial whose initial management strategy was WW. Three main findings emerged. First, WW in clinical practice was rarely purely passive: 88% of the men received some form of follow-up. Most patients underwent PSA testing and clinical visits approximately every 6 mo, regardless of risk group, whereas imaging was performed mainly in men at higher risk—and even in this group, only in approximately 15–20% of cases. Second, there were clear differences in ADT initiation between risk groups: at 18 yr, the probability of starting ADT was 6.6% (95% confidence interval [CI] 0.02–13) in men at low risk compared with 55% (95% CI 47–64) in men at high risk. Third, PSA, cT-stage, and ISUP GG at diagnosis were significantly associated with the risk of ADT initiation. For example, men with ISUP GG1, PSA 5 ng/ml, and cT1—corresponding to EAU low-risk group—had a predicted 90% ADT-free survival at 18 yr, whereas those with ISUP GG4, PSA 15 ng/ml, and cT3—corresponding to EAU high-risk group—had only 35%. Higher PSA values further reduced ADT-free survival probabilities, whereas more comorbidities slightly increased them, reflecting competing mortality. Interestingly, cT3–4 versus cT1 was not statistically associated with ADT initiation (*p* = 0.079), probably because of the small number of men in this group (*n* = 63). PSA-DT was also not associated with ADT initiation, which aligns with previous studies showing limited and inconsistent predictive value of PSA-DT for PCa outcomes [19], [20].

These results provide clinicians with data-driven insights into how clinical parameters and risk category influence the risk and timing of ADT initiation in men whose initial management strategy is WW. Patients at low risk demonstrated a very low probability of ADT initiation, a high number needed to follow up, and limited imaging use, suggesting minimal benefit from routine monitoring. In contrast, patients at high risk showed earlier and more frequent ADT initiation with a substantially lower number needed to follow up, suggesting a greater potential value of closer monitoring. These observations should support clinicians in tailoring follow-up intensity to individual patient factors such as tumor characteristics and comorbidity rather than applying a uniform approach. It should be noted that our observations relate to the frequency of follow-up visits rather than the clinical triggers for intervention, which our data did not allow us to analyze. Current EAU guidelines generally recommend imaging in case of significant PSA increases, although thresholds are not clearly defined, and ADT is generally initiated in case of symptomatic or metastatic progression or occasionally in response to a significant PSA increase [1].

Our observations are in line with the results from the nationwide, population-based cohort study by Ventimiglia et al. [21], which also showed that men with higher-risk PCa initiated ADT significantly more often than men with low-risk disease (74% vs 44% after 20 yr of follow-up). Furthermore, Albertsen et al. [22] showed that competing risks of mortality are crucial to consider in PCa management. Together, these findings support the statement that WW should not be applied uniformly but selectively—reserving closer monitoring for the patients at high risk of progression while minimizing unnecessary follow-up and harms from intervention in patients at low risk and patients with low life expectancy.

This study has several limitations. First, the retrospective selection of men into WW and AS based on predefined criteria may have led to misclassification, potentially influencing outcomes. However, this challenge reflects real-world clinical practice, where distinguishing between these strategies can be challenging and often involves a grey zone [2], [23]. Second, although ADT initiation is a clinically relevant marker of disease progression, we were unable to determine the specific reasons for starting ADT in individual patients. This lack of clearly defined or standardized triggers likely introduced variability in clinical practice and may limit the internal validity of this end point. However, this variability also reflects real-world practice, where current guidelines provide only broad recommendations and do not define clear criteria for initiating ADT in this context. Finally, this cohort was diagnosed and managed more than 20 yr ago, predating contemporary imaging, risk stratification, and treatment paradigms, which constrains generalizability. Nevertheless, the historical nature of the cohort allowed us to assess long-term outcomes over 18 yr, and although absolute risks may have shifted over time, the underlying principles and the importance of individualized follow-up remain applicable to contemporary practice.

Despite these limitations, this study has several important strengths. We used data from the Rotterdam arm of the ERSPC, a large randomized controlled trial with long-term, well-documented follow-up. The size, duration, and quality of this cohort allowed for a detailed description of WW practices and outcomes over a long period in a well-defined population. To our knowledge, this is also one of the first studies to quantify prognostic factors for ADT initiation specifically in men managed with WW and explore how these patterns might inform risk-adapted follow-up strategies.

## 5 Conclusions

These findings support a more individualized approach to WW in PCa, emphasizing that follow-up intensity should be tailored to patient risk. By reducing unnecessary visits in men at low risk and focusing on patients at higher risk, we can minimize patient and health care burden while ensuring timely intervention when progression occurs.

  ***Author contributions***: Jeroen J. Lodder had full access to all the data in the study and takes responsibility for the integrity of the data and the accuracy of the data analysis.

  *Study concept and design*: Roobol.

*Acquisition of data*: Roobol, ERSPC study group.

*Analysis and interpretation of data*: Lodder, Mulder, Remmers, van den Bergh, Bangma, Roobol.

*Drafting of the manuscript*: Lodder, Mulder.

*Critical revision of the manuscript for important intellectual content*: Lodder, Mulder, Remmers, van den Bergh, Bangma, Roobol.

*Statistical analysis*: Mulder, Remmers.

*Obtaining funding*: None.

*Administrative, technical, or material support*: None.

*Supervision*: van den Bergh, Roobol.

*Other* (specify): None.

  ***Financial disclosures:*** Jeroen J. Lodder certifies that all conflicts of interest, including specific financial interests and relationships and affiliations relevant to the subject matter or materials discussed in the manuscript (eg, employment/affiliation, grants or funding, consultancies, honoraria, stock ownership or options, expert testimony, royalties, or patents filed, received, or pending), are the following: None.

  ***Funding/Support and role of the sponsor*:** The name of the organization or organizations which had a role in sponsoring the data and material in the study are: the Dutch Cancer Society (KWF 94-869, 98-1657, 2002-277, 2006-3518, and 2010-4800), the Netherlands Organisations for Health Research and Development (ZonMW-002822820, 22000106, 50-50110-98-311, and 62300035), and the Dutch Cancer Research Foundation (SWOP), unconditional grant from Beckman-Coulter-Hybritech Inc.

**Declaration of Generative AI and AI-assisted Technologies in the Writing Process**

During the preparation of this work, the author used Microsoft Copilot to assist with language refinement and improving clarity of phrasing. After using this tool, the author carefully reviewed, edited, and verified the content as needed and takes full responsibility for the content of the publication.

## Appendix 1 ERSPC investigators

The following people are being acknowledged for their contribution to the ERSPC study.

**The Netherlands:**

Dr. W.J. Kirkels, urologist

Dr. J.B.W Rietbergen, urologist

Dr. I.W. van der Cruijsen, urologist

Dr. R. Raaijmakers, urologist

Dr. S.H. de Vries, urologist

Dr. S. Roemeling, urologist

Dr. C. Gosselaar, general practicioner

Dr. T. Wolters, urologist

Dr. R.C.N. van den Bergh, urologist

Dr. P.J. van Leeuwen, urologist

Dr. M. Bul, PhD urologist

Dr. X. Zhu, PhD urologist

Dr. L.P. Bokhorst, radiologist

Dr. A.R.Alberts, urologist

Dr. F-J. Drost, GP

Dr. J.F.M. Verbeek, Radiologist

Dr. D.F. Osses, Urologist

Dr. H.B. Luiting, Urologist

Dr. R. Hogenhout, Urologist

Drs. R.C.A. Leenen, PhD student

Drs. J. J. Lodder, PhD student

Drs. M.J. van Harten, PhD student

Drs. S.F. Westerhout, PhD student

Drs. F. Denijs, PhD student

E.F.H. Mulder, MSc student

Dr. L.D.F. Venderbos, quality of life researcher

Dr. K. Beyer, postdoctoral researcher

Dr. H.A. van Vugt

Drs. G. Yurdakul, urologist

Drs. A. Boeken-Kruger, urologist

Drs. C. Wijburg, urologist

Drs. M. Forouzanfor, urologist

Drs. M. de Boer, urologist

Dr. R. Postma, pathologist

Dr. A.N. Vis, urologist

Prof. Dr. Th van der Kwast, pathologist

Dr. R. Hoedemaeker, pathologist

Prof. Dr. A. van Leenders, pathologist

Dr. R. van Schaik, clinical chemistry

Prof. Dr. P.J. van der Maas, epidemiologist

Prof. Dr. H.J. De Koning, epidemiologist

Dr. E.A. Heijnsdijk, epidemiologist

Dr. S. Otto, epidemiologist

Dr. G. Draisma, statistician

Dr. P. Beemsterboer, epidemiologist

Dr. I. Korfage, epidemiologist

Dr. R. Boer, informatician/mathematician

Dr. M. Wildhagen, statistician

Drs. D. Nieboer, statistician

Drs. W. Merkelbach, COD committee

Dr. W. Hoekstra, COD committee

Dr. J. Blom, COD committee

Dr. T. Lock, COD committee

Dr. A. Noordzij, COC committee

Drs. RAM Damhuis, epidemiologist, cancer registry

Drs. A. Reedijk, epidemiologist, cancer registry

Dr. R. Kranse, statistician, cancer registry

Mrs. D.W. Roobol, data management

Mr. W. Roobol, website management

Mrs. E. van den Berg, administrative support

Mr. G-J de Zwart, echografist

Mrs. C.G.A.M. Franken-Raab, data management

Mrs. M. van Slooten-Midderig, data management

Mrs. A. Smit, medical assistant

Mrs. V van der Drift, data management

Mrs. E. de Bilde, data management

Mrs. M den Rooijen, data management

Mrs. L. Mani, medical assistant

Mrs. M. Visser-van Dongen, data management

Mrs. H. Versteeg-Leenheer, data management

Mrs. B. Zoutendijk, data management

Mrs. H. van Meurs, echografist

Mrs. A E. de Bruijn, secretary
