# Supplementary material for: Who, When, and How: Watchful Waiting in the ERSPC Rotterdam
Source: Eur Urol Open Sci. 2026 Apr 2;87:40–7. doi: 10.1016/j.euros.2026.03.013 (PMC13084745; doi:10.1016/j.euros.2026.03.013)
Supplement: Supplementary Data 1 [file mmc1.docx]

**Supplementary material**

**Supplementary Table 1**. Baseline characteristics of WW and AS group.

|  |  | WW (N = 537) | AS (N = 1022) |
| --- | --- | --- | --- |
| Age |  |  |  |
|  | Median (IQR) | 79 (74 – 83) | 72 (68 – 74) |
| PSA |  |  |  |
|  | Median (IQR) | 12 (5.7 – 22) | 4.5 (3.2 – 6.9) |
|  | Missing | 22 | 31 |
| Year of diagnosis |  |  |  |
|  | Median (IQR) | 2009 (2004 – 2015) | 2007 (2002 – 2010) |
| cT-stage |  |  |  |
|  | T1, n (%) | 315 (61%) | 865 (85%) |
|  | T2, n (%) | 142 (27%) | 146 (14%) |
|  | T3, n (%) | 55 (11%) | 4 (0.39%) |
|  | T4, n (%) | 8 (1.5%) | 0 (0%) |
|  | Missing, n | 17 | 7 |
| Screening arm |  |  |  |
|  | Screening, n (%) | 249 (46%) | 738 (72%) |
|  | Control, n (%) | 288 (54%) | 284 (28%) |
| Grade group |  |  |  |
|  | 1, n (%) | 231 (46%) | 1018 (100%) |
|  | 2, n (%) | 159 (32%) | 0 (0%) |
|  | 3, n (%) | 48 (9.6%) | 0 (0%) |
|  | 4, n (%) | 36 (7.2%) | 0 (0%) |
|  | 5, n (%) | 26 (5.2%) | 0 (0%) |
|  | Missing, n | 37 | 4 |
| Start on ADT |  |  |  |
|  | Yes, n (%) | 173 (32%) | 114 (11%) |
|  | No, n (%) | 364 (68%) | 908 (89%) |
| Comorbidities |  |  |  |
|  | 0, n (%) | 176 (33%) | 467 (46%) |
|  | 1, n (%) | 174 (32%) | 339 (33%) |
|  | 2, n (%) | 110 (20%) | 152 (15%) |
|  | 3+, n (%) | 77 (14%) | 64 (6.3%) |

ADT = androgen deprivation therapy; AS = active surveillance; cT-stage = clinical T-stage; PSA = prostate specific antigen; WW = watchful waiting.

**
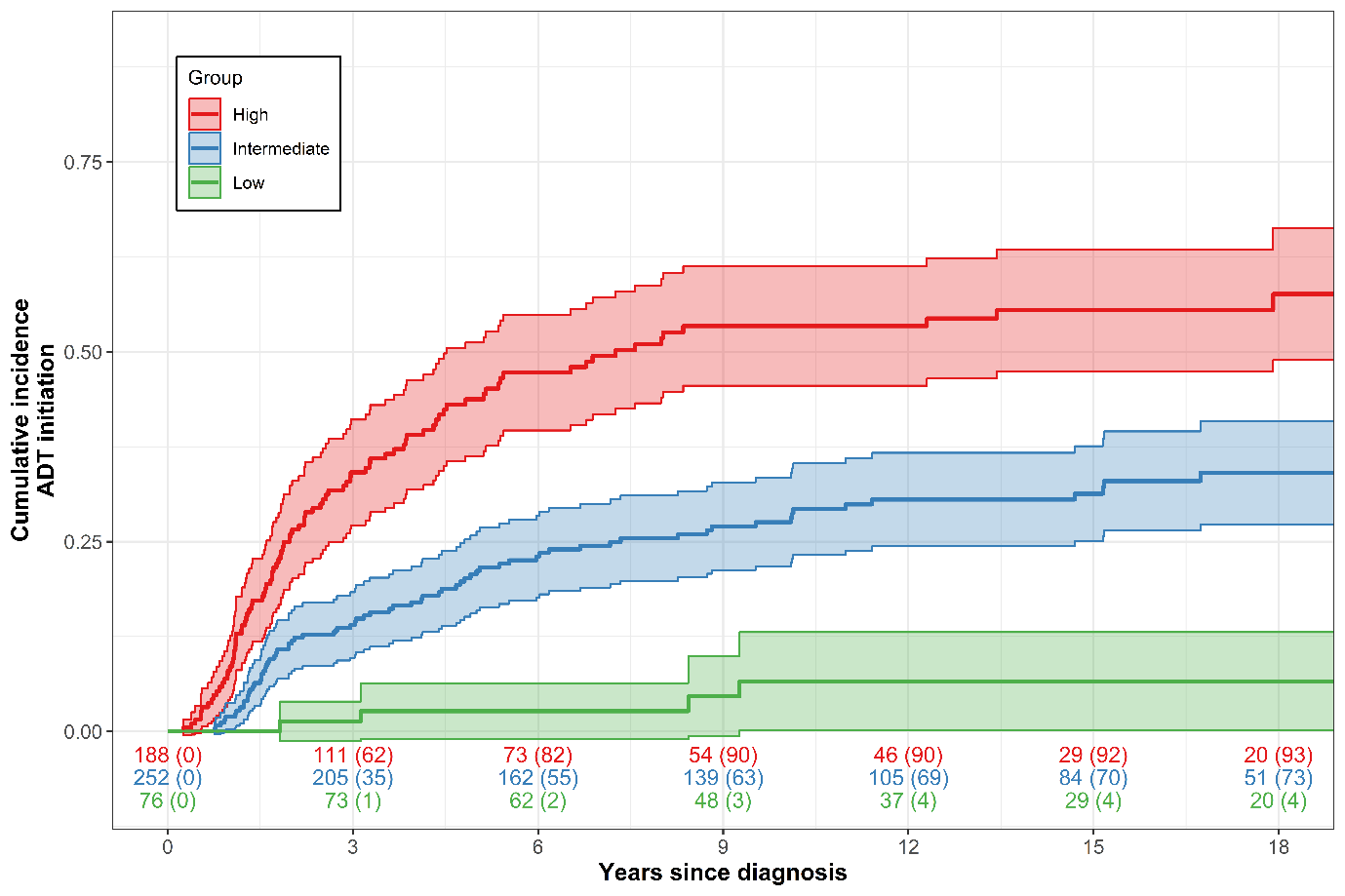
**

**Supplementary Figure 1.** Cumulative incidence of ADT-initiation over time across risk groups. ADT = Androgen Deprivation Therapy.
